# 

**Published:** 1890-03

**Authors:** 


### CONTENTS.

### COMMUNICATIONS.

The Teeth and Oral Cavity of Pregnant Women ........ 109
Changes of the Oral Secretions ........ 117
Evolution in Dentistry ........ 123
How to Stand ........ 127

### SELECTIONS.

Mouth-Breathing and the Teeth ........ 129
Excision of the Facial Bones ........ 130
Dangers of Celluloid ........ 132
Menthol in Acute Rhinitis, etc ........ 133
Local Anæsthesia by Cocaine ........ 134
Mind Blindness ........ 135
A Leech in the Larynx ........ 136
The Red Line Along the Gums ........ 137
A Simple Remedy for Thrush and Sordes ........ 138
Statistics of Breathing ........ 139
Miscellaneous Items ........ 140–143
The Exciting Cause of Tetanus ........ 144
International Medical Congress ........ 146

### CORRESPONDENCE.

Letter from Rome, Italy ........ 150
Letter from Budapest, Hungary ........ 151
Letter from London, England ........ 154

### EDITORIAL.

The Dental Protective Association ........ 156
"Is it a Fraud?" ........ 157
International Medical Congress—Dental Section ........ 158

